# Antimicrobial efficacy and cytotoxicity of ethanolic extracts of *Salvia officinalis* and *Juglans regia* against oral pathogens

**DOI:** 10.34172/japid.025.3710

**Published:** 2025-02-16

**Authors:** Sara Ghadaksaz, Safar Farajnia, Fatemeh Pournaghiazar, Hamed Ebrahimzadeh Leylabadlo, Reza Salahlou, Abbas Delazar, Amir Zandesh

**Affiliations:** ^1^Dental and Periodontal Research Center,Tabriz University of Medical Sciences, Tabriz, Iran; ^2^Biotechnology Research Center, Tabriz University of Medical Sciences, Tabriz, Iran; ^3^Department of Operative Dentistry, Faculty of Dentistry, Tabriz University of Medical Sciences, Tabriz, Iran; ^4^Liver and Gastrointestinal Diseases Research Center, Tabriz University of Medical Sciences, Tabriz, Iran; ^5^Drug Applied Research Center, Tabriz University of Medical Sciences, Tabriz, Iran

**Keywords:** Candida albicans, Dental caries, Dentistry, Lactobacillus casei, Oral hygiene, Periodontal diseases, Streptococcus mutans

## Abstract

**Background.:**

Herbal extracts have gained attention for their potential benefits in promoting oral health and preventing dental caries and periodontal diseases. This study evaluated the antimicrobial effects and cytotoxicity of ethanolic extracts of *Salvia officinalis *and* Juglans regia*, both individually and in combination, against *Streptococcus mutans, Lactobacillus casei, *and* Candida albicans*, microorganisms associated with oral diseases.

**Methods.:**

In this *in vitro* study, the hydroalcoholic (ethanolic) extracts of *J. regia *and *S. officinalis* were prepared using the maceration method. To determine the antimicrobial effectiveness, the zone of inhibition in the disk agar diffusion, minimum inhibitory concentration (MIC) and minimum bactericidal concentration (MBC) were assessed for each extract, separately and in combination. The extracts’ cytotoxicity was investigated at their effective doses using the MTT (methyl thiazole tetrazolium) assay.

**Results.:**

Compared to the negative control, ethanolic extracts of *S. officinalis *and* J. regia* exhibited a significant inhibitory effect (*P*<0.001) on *S. mutans *and *L. casei. Salvia officinalis* extract exhibited antimicrobial activities, as evidenced by the MIC values of 237.5 µg/mL for *S. mutans*, 118.7 µg/mL for *Lactobacillus casei*, and 31.25 mg/mL for *C. albicans*. The ethanolic extract of *Juglans regia* exhibited MIC values of 29.6 µg/mL for *S. mutans*, 475 µg/mL for *L. casei*, and 15.62 mg/mL for *C. albicans*, respectively. MTT assay results showed that the extracts had no cytotoxic effects at the MIC on the L929 cell line; however, pure chlorhexidine was toxic at 0.2% concentration.

**Conclusion.:**

The study results revealed that the ethanolic extracts of *S. officinalis *and* J. regia* and their combined application showed antimicrobial activity against pathogenic microorganisms pertinent to oral health. In addition, cytotoxicity evaluations indicated that these extracts are non-toxic to the L929 cell line at effective concentrations.

## Introduction

 The oral microbiome, characterized by its dynamic and polymicrobial nature, is a fundamental precursor to periodontitis (PD) and dental caries, ranking high among the most common microbially induced diseases globally. As these microbial communities evolve, the metabolic activities of the microorganisms, coupled with the by-products generated by the host immune response, induce alterations in the local environment. These environmental changes promote the proliferation or increased representation of specific microorganisms associated with a dysbiotic state, contributing to the pathogenesis of oral diseases.^1,2^ PD is a long-lasting inflammatory state that damages the periodontal ligaments surrounding the teeth. Standard nonsurgical procedures, such as scaling and root planing (SRP), often need additional therapies to improve effectiveness, as they do not always completely eliminate pathogens.^3^ Dental caries (tooth decay) occurs through a natural mechanism in which bacteria in the oral biofilm cause oscillations in pH, leading to enamel demineralization and the formation of apparent lesions.^4^ Caries, if left untreated, can cause pain and abscess and may even lead to systemic infections, as well as functional or psychosocial impairment.^5^ The correlation between PD and dental caries has been a topic of debate in recent years. Some studies have indicated an inverse relationship, while others have found a positive correlation between these two diseases.^6,7^ Also, a recent systematic review demonstrated that the two disorders are interconnected through bidirectional relationships. This finding underscores the complexity of their interaction so managing the pathological microbial environment in the oral cavity is essential for controlling both periodontal diseases and dental caries, as it helps to prevent the progression and exacerbation of these common oral diseases.^8^

 Controlling dental biofilm is achieved through various methods, including using mouth rinses containing chlorhexidine gluconate (CHX) as a major ingredient. CHX is utilized for its potential effectiveness in managing oral soft tissue diseases, PD, plaque-induced gingivitis, and dental caries. Additionally, repeated use of this compound may result in side effects such as tooth discolorations, loss of taste perception, and the potential development of antimicrobial resistance (AMR).^9,10^ However, natural products have demonstrated biological compatibility and possess therapeutic benefits.^11^ For that reason, the development of alternative biologically active antimicrobial natural agents capable of acting synergistically against oral microbial biofilm, which causes dental carious process and periodontal diseases, is essential.^12^ That is why plant-derived extracts or bioactive substances are considered a desirable alternative to routine antibiotics and synthetic agents with less side effects and also, to overcome the crisis of AMR.^13^

*Juglans regia*, commonly known as Iranian walnut, belongs to the *Juglandaceae *family and is widely found worldwide. Different parts of the plant have significant therapeutic uses.^14^ The leaves are easily available in large quantities and serves as a source of health care compounds. They are used in indigenous medicine to treat hypoglycemia, diarrhea, chronic venous insufficiency, hemorrhoids, and microbial or fungal infections.^15^*J. regia* stem is used as an anti-diarrhea and anti-parasitic agent, and the dried trunk bark is traditionally used as a toothbrush in some countries.^16^ Some in vitro studies have demonstrated the biological properties of *J. regia* extract, including anti-proliferative, anti-inflammatory properties, and broad-spectrum antimicrobial activity; it prevents the growth of several types of pathogenic microorganisms.^17-19^

*Salvia officinalis *L. (Sage) is an aromatic perennial shrub of the *Labiatae/ Lamiaceae* family. Sage is the largest species of this family. The plants of this family are found all around the world, and the *S. officinalis *species is indigenous to the Mediterranean and Middle East regions. It has become natural worldwide, especially in North America and Europe.^20,21^ Recent studies have been executed to document the traditional applications and investigate new biological properties of different parts of this plant. These research studies have shown an extensive range of medicinal activities, including anti-cancer, anti-inflammatory, analgesic, anti-mutagenic, antioxidant, antimicrobial, anti-dementia, glucose lowering, and blood lipid-lowering effects. Ethanolic extract and essential oil of *S. officinalis* have strong antibacterial and bacteriostatic effects against gram-negative and gram-positive pathogens. Among gram-positive bacteria, *Bacillus cereus*, *Bacillus subtilis*, *Bacillus megaterium*, *Staphylococcus epidermidis*, *Enterococcus faecalis*, and *Listeria monocytogenes *show high susceptibility to this extract. The effects of *S. officinalis* on gram-negative bacteria depend on the type of extraction method used, and *S. officinalis* essential oil has a considerable inhibitory effect on the growth of *Escherichia coli, Klebsiella pneumoniae*,* Klebsiella oxytoca*,* Pseudomonas morgana*, *Aeromonas sobria*,* Aeromonas hydrophila*, *Salmonella anatoma*,* Salmonella typhi*, *Salmonella enteritidis*, and *Shigella sonnei*. This plant’s broad antibacterial and antifungal effect on a wide range of microorganisms such as *Pseudomonas*, *Aspergillus*, and *Candida* has been reported.^22-24^

 The current study aimed to determine the antimicrobial properties of *J. regia* and *S. officinalis *ethanolic extracts and their combination on oral pathogenic species, namely *Streptococcus mutans*, *Lactobacillus casei, *and *Candida albicans,* compared to 0.2% chlorhexidine (Behsa, Iran). Additionally, the cytotoxicity of substances was evaluated using the MTT assay. Considering this information, *J. regia* bark and *S. officinalis *aerial parts may have the potential for application in oral healthcare products.

## Methods

 This in vitro study was conducted at the Biotechnology Research Center, Tabriz University of Medical Sciences, from October 2023 to August 2024. The Ethics Committee of the Research and Technology Vice-Chancellor of Tabriz University of Medical Sciences approval code is IR.TBZMED.VCR.REC.1402.117.

###  Preparation of extracts

 Bark of *J. regia* and the aerial parts of *S. officinalis* were purchased from local markets in Tabriz (Iran) and identified by a pharmacologist. Extraction was performed by maceration technique by soaking 200 g of fine powder in 2000 mL of ethanol for each plant species. After covering the heads of the *Erlenmeyer flask* with aluminum foil, it was placed on a shaker at 90 rpm for 48 hours (Heidolph Unimax, Germany). After the solvent and the plant were homogenized, the solutions were filtered by 0.5-mm Watman filter paper (Watman, USA) and placed in a rotary evaporator (Heidolph WD 2000, Germany) to separate the solvent from the extract. The obtained pure extract was stored in the refrigerator (-18 ºC) in sterile vials for the microbial and cytotoxicity tests.

###  Preparation of microbial species

*Streptococcus mutans *(PTCC: 1683),* L. casei *(PTCC: 1608),and* C. albicans* (PTCC: 5027) standard microbial strains were prepared in lyophilized form from the Persian Type Culture Collection (PTCC, Tehran, Iran) reference center. To prepare bacteria from lyophilized samples, first, the samples were cultured in a liquid broth culture medium overnight at 35 ºC. After creating turbidity in the liquid broth culture medium, to ensure purity, the samples were isolated on the solid culture medium [BHI agar (Spain, CONDA) for *C. albicans *and* S. mutans *andMRS agar (Germany, Merck) for *L. casei*].

###  Determination of antimicrobial effects using the agar disk diffusion method

 Antimicrobial activity was assessed using the agar disk diffusion method explained by Finegold and Mart, 1982.^22^ Each extract was prepared in three concentrations of 1000, 500, and 250 mg/mL. Then, blank paper disks (6.4-mm blank paper disks) were loaded with 30 µL. Also, discs impregnated with chlorhexidine (positive control) and ethanol (negative control) were prepared as control groups. The specific culture medium of different strains of microorganisms was cultivated using a sterile cotton swab with suspensions containing 0.5 McFarland, and discs containing the extract were placed on all prepared plates and kept at 37 ºC for 24 hours. The diameter of the inhibited areas was measured and recorded as the average diameter of complete growth inhibition.^25-27^

###  Determining the minimum inhibitory concentration

 To measure the minimum inhibitory concentration (MIC) for each of *J. regia* and *S. officinalis* extracts and their combination, 14 different concentrations in the range of 250 mg/mL to 29.6 µg/mL were prepared using broth medium according to the broth microdilution method (CLSI M07 protocol). An 0.5 McFarland (1.5 × 10^8^ CFU/mL) suspension was prepared for each microbial species in the test tube. Then, the MIC was investigated in a 96-well microplate. Chlorhexidine was used as a positive control, and ethanol was used as a negative control. After incubation, the growth or non-growth of microorganisms was evaluated by checking the turbidity in the wells. The concentration of the first well in which no growth was observed was the MIC of the microorganism growth by that extract.

###  Determining the minimum bactericidal concentration

 The contents of the microplate wells in which the MIC determination test was performed (after recording the results) were transferred to the surface of the nutrient agar culture medium by a sampler comprising 10 µL and cultivated. Then, the plates were incubated at 37ºC for 24 hours. After this period of incubation time, the minimum concentration of the well that led to the inhibition of bacterial growth on the surface of the nutrient agar plate was assessed as the minimum bactericidal concentration (MBC).^28,29^

###  Evaluation of cytotoxicity 

 The MTT assay (methylthiazol tetrazolium) method was used to investigate the cytotoxic effects of the samples. In 96‐well cell culture plates, the L929 fibroblasts were exposed to different concentrations of *J. regia *and* S. officinalis *(1/2 MIC, 1 MIC, and 2 MIC) and chlorhexidine mouthwash (1, 10, and 100%) for 1 min and 5 min, at 37 ºC. Finally, the absorbance of the plates was read at 570 nm through an ELISA plate reader.

###  Statistical analysis

 The results were reported as descriptive statistics (Mean ± SD). One-way analysis of variance (ANOVA) was used to compare the groups. SPSS software (version 25, USA) was used for data analysis. The significance level was defined at < 0.05.

## Results

###  Disk diffusion 

Tables 1 and 2 present the inhibition zones due to* S. officinalis *and *J. regia*ethanolic extracts and their combination in three concentrations (1000, 500, and 250 mg/mL), negative control (70% ethanol) and positive control (0.2% chlorhexidine).

**Figure 1:**
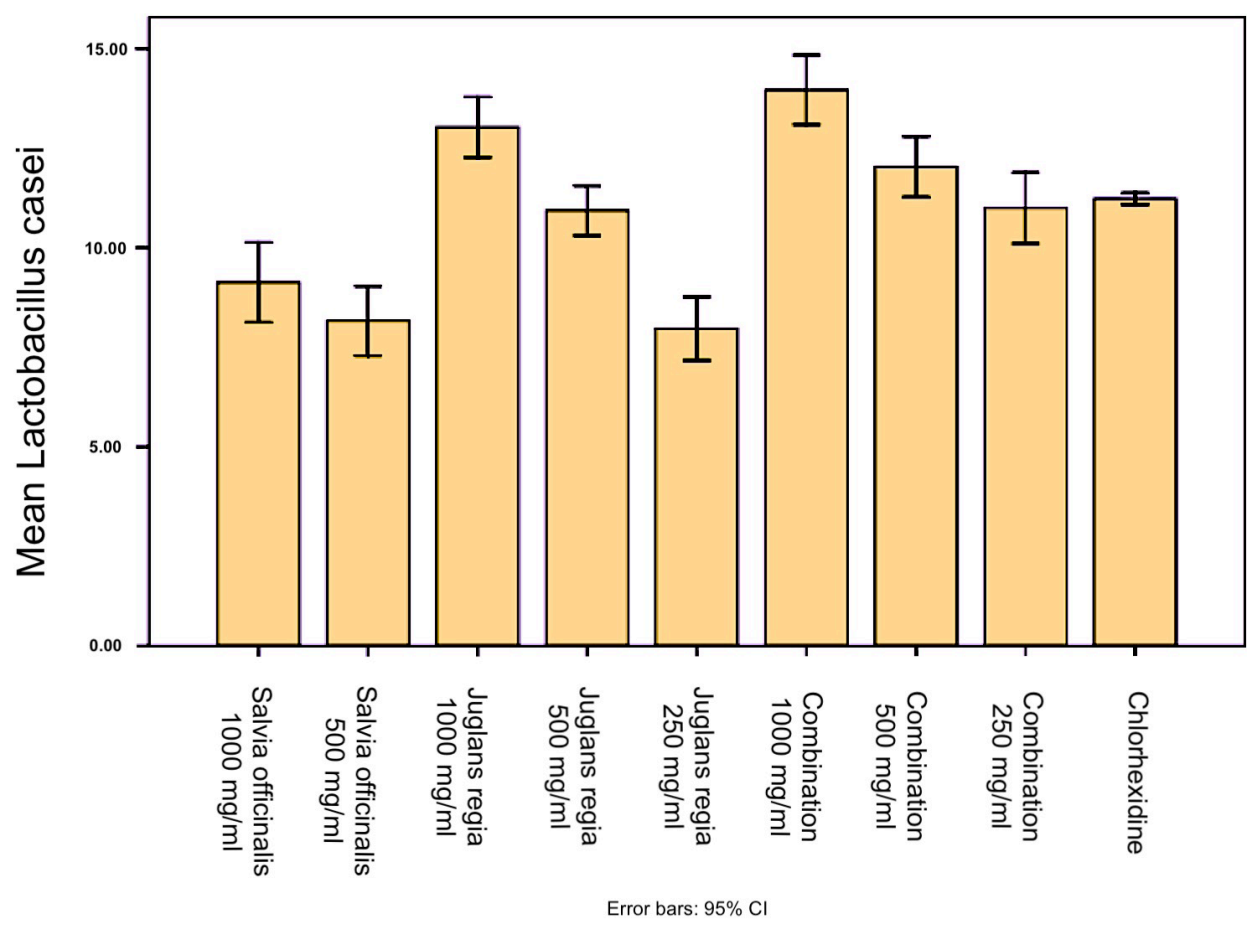
The Results of the Disk Diffusion Method for* Lactobacillus casei.* Column charts and error bars represent means ± SD

**Figure 2:**
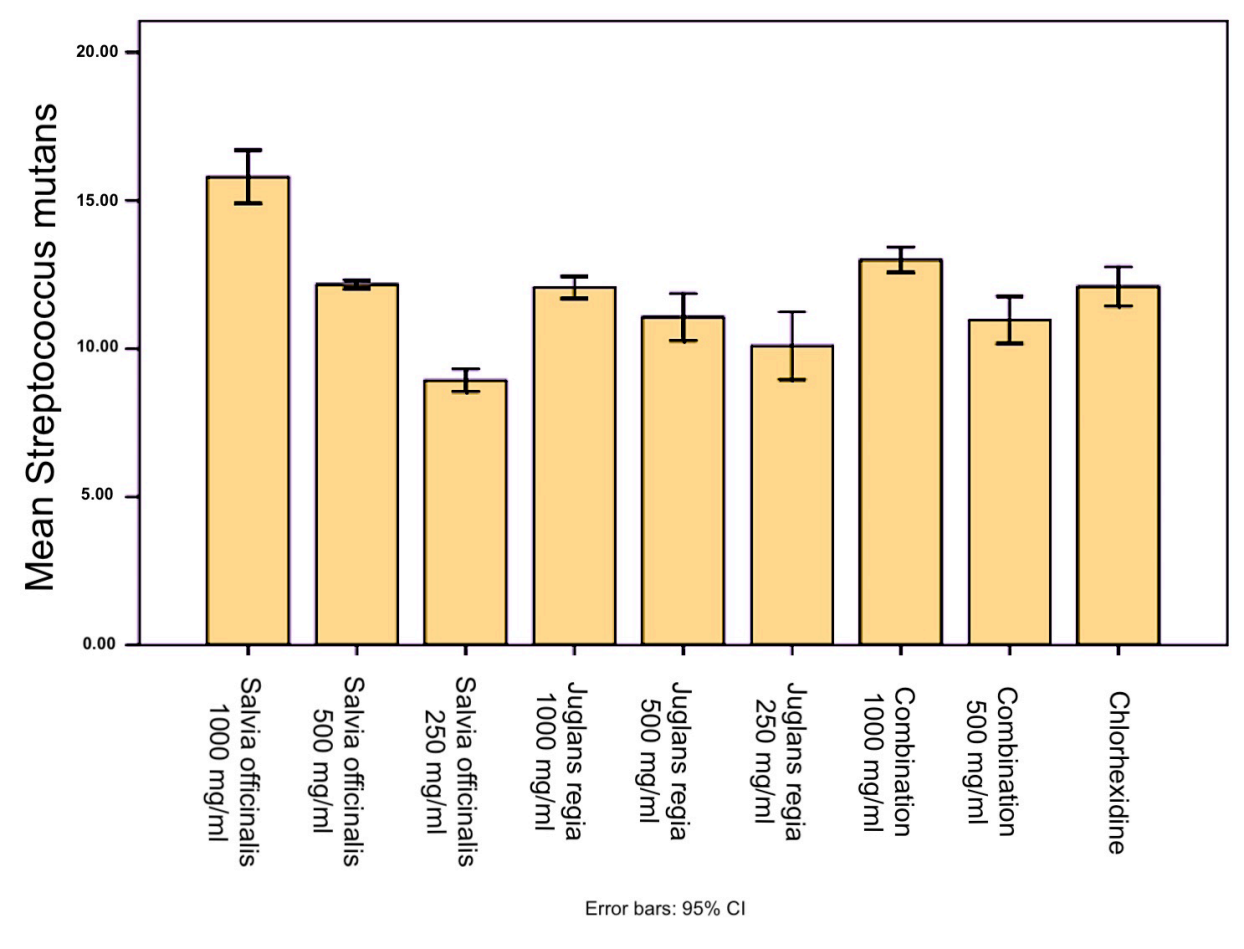
The Results of the Disk Diffusion Method for* Streptococcus mutans.* Column charts and error bars represent means ± SD

**Figure 3:**
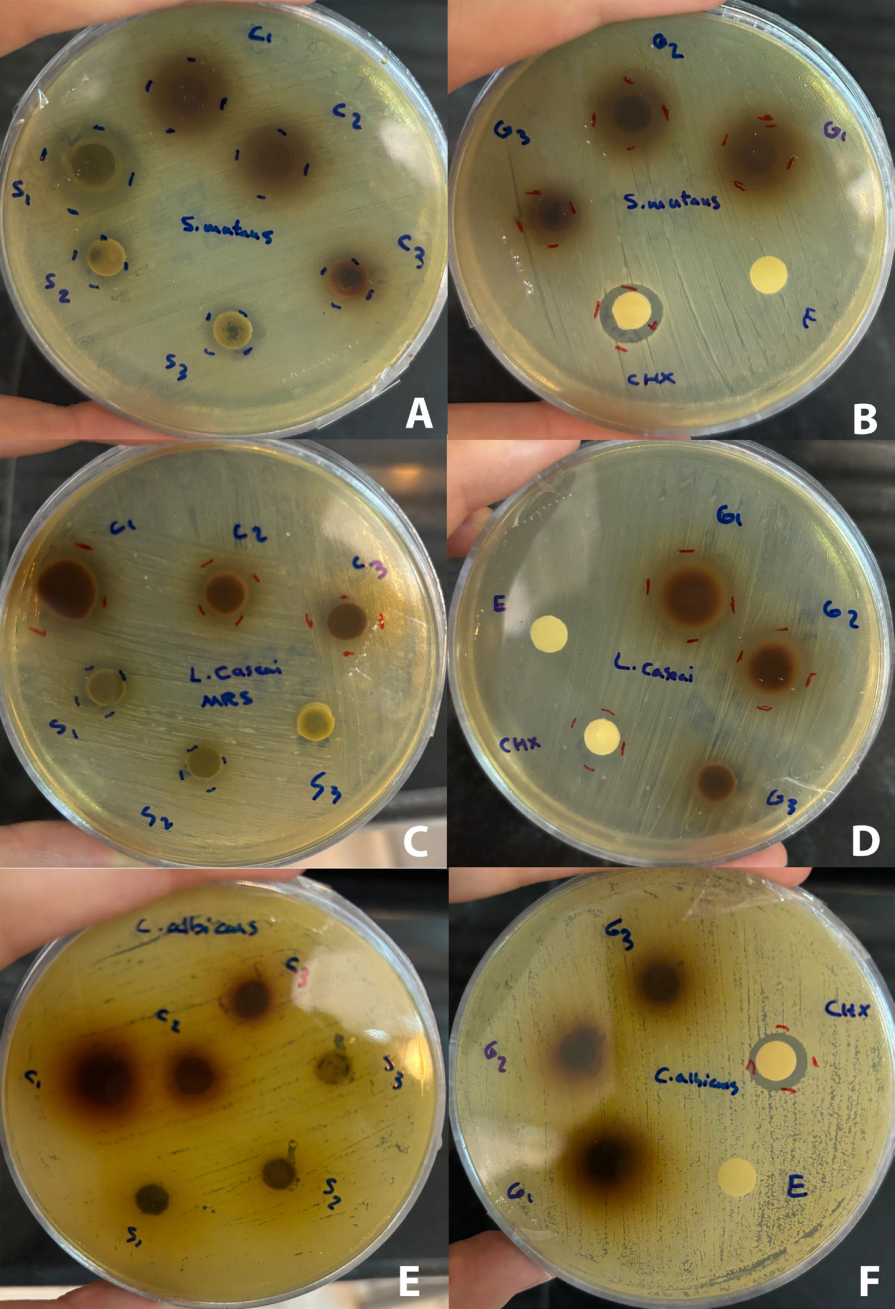
The Results of the Disk Diffusion Method for Extracts. Zones of inhibition of* Streptococcus mutans *(A and B), *Lactobacillus casei *(C and D), and *Candida albicans* (E and F). “R” stands for *Juglans regia *extract, “S” stands for *Salvia officinalis* extract, “C” stands for the combination of *Juglans regia *and *Salvia officinalis* extract, “E” stands for 70% ethanol, “CHX” stands for chlorhexidine. Different concentrations of extractsin mg/mL: 1:1000, 2:500, 3:250

 According to the results, *S. officinalis* exhibited a significant inhibitory effect (*P* < 0.001) on *Streptococcus mutans* and *L. casei* species compared to the negative control. Also, the inhibition zone of *Salvia officinalis* (1000 mg/mL) against *S. mutans* was significantly (*P* < 0.0001) larger than the inhibition zone of chlorhexidine (0.2%). *Juglans regia*exerted a significant inhibitory effect (*P* < 0.001) on *S. mutans* and *L. casei* species compared to the negative control. However, none of the extracts and their combination showed an inhibitory zone against *C. albicans* (Figures 1 to 3).

###  MIC and MBC tests

**Figure 4:**
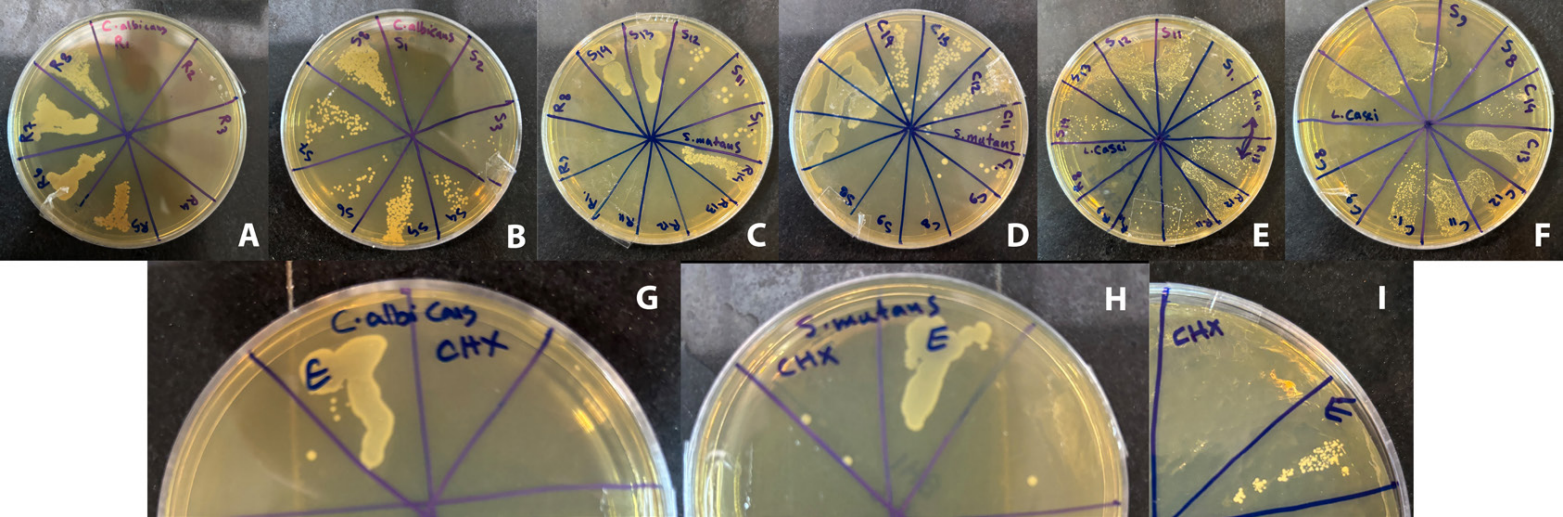
The Results of the MBC forExtracts. MBC results of *Candida albicans *(A, B, and G), Streptococcus mutans (C, D, and H), and Lactobacillus casei (E, F, and I). “R” stands for *Juglans regia *extract, “S” stands for *Salvia officinalis* extract, “C” stands for the combination of *Juglans regia* and* Salvia officinalis* extract, “E” stands for ethanol, “CHX” stands for chlorhexidine. Different concentrations of extractsin μg/mL: 1: 250 × 10^3^, 2: 125 × 10^3^, 3: 62.5 × 10^3^, 4: 31.25 × 10^3^, 5: 15.62 × 10^3^, 6: 7.81 × 10^3^, 7: 3.9 × 10^3^, 8: 1.9 × 10^3^, 9:0.95 × 10^3^, 10: 475, 11: 237.5, 12: 118.7, 13: 59.3, 14: 29.6

 MIC and MBC results showed that *Juglans regia* bark ethanolic extract exerted the strongest inhibitory and bactericidal effects on *S. mutans* with an MIC of 29.6 µg/mL. The ethanolic extract of *S. officinalis *had the lowest MIC (118.7 µg/mL) on *L. casei*. In addition, *C. albicans* showed less sensitivity to the extracts. However, no synergistic effect was observed in a combination of the two extracts (Figure 4). The MIC and MBC were evaluated for antimicrobial activity, and the results are summarized in Tables 3 and 4.

**Table 1:** The results of the disk diffusion method for* Lactobacillus casei*

**Organism**	**Sample**	**Diameter of inhibition zone (mm)**	***P*** ** value**
*Lactobacillus casei*	Salvia officinalis 1000 mg/mL	9.13 ± 0.40	< 0.001
	Salvia officinalis 500 mg/mL	8.16 ± 0.35	
	Juglans regia 1000 mg/mL	13.03 ± 0.30	
	Juglans regia 500 mg/mL	10.93 ± 0.25	
	Juglans regia 250 mg/mL	7.96 ± 0.32	
	Combination 1000 mg/mL	13.96 ± 0.35	
	Combination 500 mg/mL	12.03 ± 0.30	
	Combination 250 mg/mL	11.00 ± 0.36	
	Chlorhexidine	11.23 ± .057	

**Table 2:** The results of the disk diffusion method for *Streptococcus mutans*

**Organism**	**Samples**	**Diameter of Inhibition Zone (mm)**	***P*** ** value**
*Streptococcus mutans*	*Salvia officinalis* 1000 mg/mL	15.80 ± 0.36	< 0.001
	*Salvia officinalis* 500 mg/mL	12.16 ± 0.05	
	*Salvia officinalis* 250 mg/mL	8.93 ± 0.15	
	*Juglans regia* 1000 mg/mL	12.06 ± 0.15	
	*Juglans regia* 500 mg/mL	11.06 ± 0.32	
	*Juglans regia* 250 mg/mL	10.10 ± 0.45	
	Combination 1000 mg/mL	13.00 ± 0.17	
	Combination 500 mg/mL	10.96 ± 0.32	
	Chlorhexidine	12.10 ± 0.26	

**Table 3:** MIC results for the samples against the selected organisms

**Extracts**	***Streptococcus mutans***	***Lactobacillus casei***	***Candida albicans***
*Salvia officinalis*	237.5 µg/mL	118.7 µg/mL	31.25 × 10^3^ µg/mL
*Juglans regia*	29.6 µg/mL	475 µg/mL	15.62 × 10^3^ µg/mL
Combination	118.7 µg/mL	237.5 µg/mL	15.62 × 10^3^ µg/mL
Ethanol	+	+	+
chlorhexidine	-	-	-
Broth + microorganism	+	+	+
Extract + broth	-

**Table 4:** MBC results for the samples against the selected organisms

**Extracts**	***Streptococcus mutans***	***Lactobacillus casei***	***Candida albicans***
*Salvia officinalis*	0.95 × 10^3^ µg/mL	475 µg/mL	62.5 × 10^3^ µg/mL
*Juglans regia*	29.6 µg/mL	0.95 × 10^3^ µg/mL	31.25 × 10^3^ µg/mL
Combination	237 µg/mL	0.95 × 10^3^ µg/mL	31.25 × 10^3^ µg/mL
Ethanol	+	+	+
chlorhexidine	-	-	-
Broth + microorganism	+	+	+
Extract + broth	-

###  Cytotoxicity test

 Based on the cytotoxicity test results on the L929 cell line, the extracts and their combination had no toxic effect in their MIC concentration on *S. mutans* compared to the positive control. However, chlorhexidine (0.2%) in pure commercial concentration and diluted to 10% showed cytotoxic effects, but the diluted concentration of 1% chlorhexidine had no toxicity compared to the positive control.

## Discussion

 In the present study, disk diffusion results showed that the ethanolic extract of *S. officinalis* exhibited a significant inhibitory effect on bacterial species *S. mutans* and *L. casei *compared to the negative control, the inhibition zone of *S. officinalis *(1000 mg/mL) against *S. mutans* was significantly larger than chlorhexidine. The *S. officinalis* MIC values for *S. mutans* and *L. casei* were 237.5 and 118.7 µg/mL, respectively. The *J. regia *MIC values for *S. mutans* and *L. casei* were 29.6 and 475 µg/mL, respectively. The* S. officinalis* and *J. regia *MIC values for *Candida albicans* were 31.25 and 15.62 mg/mL, which indicates the lower sensitivity of *C. albicans* compared to the other two studied species.

 According to Kermanshah et al,^30^ *S. officinalis* has a growth inhibitory effect against *S. mutans*, *Actinomyces viscose,* and* Lactobacillus rhamnosus*. The MIC and MBC of hydroalcoholic extract of *S. officinalis* on *S. mutans* were 6.25 and 50 µg/mL, respectively. Comparison between the results indicates that, despite similar test conditions in the above study and ours, Kermanshah et al observed stronger antimicrobial effects of the *S. officinalis* hydroalcoholic extract. This discrepancy may be attributed to variations in the concentration of the plant’s active compounds, likely resulting from growth under different environmental conditions.

 Sookto et al^31^ investigated the anticandidal activities of *S. officinalis* against *C. albicans*. *S. officinalis* extract showed anticandidal activity against the* C. albicans* strain with an inhibition zone of 19.5‒40.5 mm.

 A previous study highlighted a notable improvement in bleeding on probing parameter (BOP) within the test group that used *S. officinalis*. The results showed that using *S. officinalis *gel in conjunction with SRP significantly enhanced clinical periodontal parameters. These findings indicated that *S. officinalis*, due to its phytochemical activities, provided a potent and secure substitute to synthetic pharmaceutical agents for the management and treatment of PD.^3^

 Based on previous studies, the antimicrobial properties of *S. officinalis* are associated with the terpenes and terpenoid compounds found in this plant,^24^ Additionally, a study on* S. officinalis* identified and characterized three key multicyclic terpenoids—ferruginol, sugiol, and sclareol—which demonstrated remarkable antimicrobial properties.^32^ Ursolic acid and oleanolic acid, two triterpenoids of *S. officinalis*, have an inhibitory effect on the growth of multidrug-resistant bacteria such as penicillin-resistant* Streptococcus pneumoniae*,methicillin-resistant* S. aureus*, and vancomycin-resistant* enterococci*. The effect of ursolic acid on multi-drug-resistant bacteria and *Enterococcus faecium* is stronger than ampicillin.^33^ In addition, the antibacterial and antiviral effects of this extract have been attributed to compounds such as alpha,beta-thujone, and 1-8 cineol, as well as flavonoids such as apigenin. In addition, linalool, Borneol, and alpha and beta-caryophyllene compounds are also found in *S. officinalis*.^34^

 In a study conducted in 2019, the anti-*L. casei* and anti-*S. mutans* properties of glass ionomer restorative cement (GIC) modified with *S. officinalis* extract powder were deemed safe and effective. The mean diameter of the inhibitory zones of the experimental and control groups showed a significant difference in *S. mutans* and *L. casei*, indicating the inhibitory activities of glass ionomer restorative containing *S. officinalis* against the selected bacteria by dose-response method.^35^

 The results of disk diffusion in the present research showed that the ethanolic extract of walnut trunk has an inhibitory effect on *L. casei* and *S. mutans* bacteria. These values ​​are comparable with the inhibition halo created by chlorhexidine on these two bacterial strains.

 In traditional medicine, the use of various parts of the walnut tree* (J. regia)* to clean the teeth and oral hygiene has been considered. Nancy et al^36^ showed that the *J. regia *bark extract could be effective against cariogenic bacteria such as *S. mutans*, *Streptococcus sobrinus*, and *A. viscosus.* Phytochemical tests showed the presence of polyphenols, alkaloids, steroids, saponins, and tannins, and compounds rich in polyphenols and tannins in this plant prevented the growth of various microorganisms.^37^

 Furthermore, findings from a recent study indicated that the bark of *J. regia* comprises juglone, which is its most significant component. Juglone has been identified as an effective agent with antibacterial, antifungal, antiplaque, anti-cariogenic, and tooth-whitening properties.^38^

 Zakavi et al^19^ investigated the antimicrobial effects of aqueous and ethanolic extracts of *J. regia *bark on four different oral bacteria: *S. aureus, Streptococcus salivarius, Streptococcus sanguis, *and* S. mutans*. The antibacterial effect of *J. regia* trunk extract was evaluated in a dose-dependent manner.

 A previous study indicated that the aqueous extract of *J. regia* leaves is effective in inhibiting the growth of gram-positive bacteria (*B. subtilis, B. cereus, *and *S. aureus*); however, gram-negative species (*E. coli, K. pneumoniae, *and *P. aeruginosa*) and fungi (*C. albicans *and* C. neoformans*) are resistant to this extract. The results indicated that the walnut tree leaves exhibited more limited antimicrobial activity than the trunk. In the *J. regia* plant, *flavonoids* showed antimicrobial properties, and *quercetin* and other related compounds acted mainly by inhibiting the DNA gyrase enzyme.^39^

 Moreover, another study confirmed the therapeutic potential of *J. regia* extracts, attributed to the significant antimicrobial effect of its bark against *Escherichia coli*, *Staphylococcus*, *Enterobacter*, and *Pseudomonas*. These findings suggest the potential for incorporating *J. regia* bark into oral care practice to prevent periodontal diseases and dental caries.^40^

 Another study also showed that *J. regia* bark extract significantly inhibited the growth of oral bacteria compared to standard mouthwashes such as chlorhexidine and Listerine. The inhibition zone *of J. regia* bark ethanolic extracts against cariogenic bacteria showed a larger inhibition zone with an average of 7.96 mm, and the distilled water extract of *J. regia* bark showed a lower inhibition zone with an average of 6.36 mm compared to the values reported in the present study.^11^

 This research revealed that ethanolic extracts of *J. regia* bark and aerial parts of *S. officinalis* significantly inhibited the growth of the tested oral pathogenic microorganisms, and those reports are consistent with our findings. Despite these promising observations, this research has some limitations that require consideration. Microbial susceptibility tests only evaluate in vitro antimicrobial activities; therefore, it cannot be assured that these antimicrobial agents will be effective in clinical applications. Moreover, these tests do not provide insights into the resistance mechanisms in pathogens.

## Conclusion

 The results of the present study indicated that ethanolic extracts of aerial parts of *S. officinalis* and *J. regia* bark exerted extensive antibacterial effects on *S. mutans* and *L. casei*. Also, the strongest MIC and killing effect (MBC) was seen in the main bacteria known in dental caries, *S. mutans* by* J. regia* bark. The growth inhibitory effects of these extracts on *C. albicans* were limited, and they were only effective at high concentrations. Cytotoxicity studies showed that these extracts had no toxic effect on L929 cells at effective concentrations. Given the clinical importance of preventing caries and periodontal diseases and reducing pathogenic bacteria in the oral environment, these extracts can be used in oral and dental health products as mouthwashes, gels, and dressings in clinical practice. Supplementary in vivo studies may be needed to investigate the antimicrobial and cytotoxicity activities of synthetic nanoparticles of the extracts.

## Competing Interests

 The authors deny any conflicts of interest related to this study.

## Consent for Publication

 Not applicable.

## Data Availability Statemen

 All data regarding the methodology of the manuscript have been shared.

## Ethical Approval

 The present study was approved by the Ethics Committee of Tabriz University of Medical Sciences under the code IR.TBZMED.VCR.REC.1402.117.
